# Inhibiting Ferroptosis Prevents the Progression of Steatotic Liver Disease in Obese Mice

**DOI:** 10.3390/antiox13111336

**Published:** 2024-10-31

**Authors:** Gi Cheol Park, Soo-Young Bang, Ji Min Kim, Sung-Chan Shin, Yong-il Cheon, Kwang Min Kim, Hanaro Park, Eui-Suk Sung, Minhyung Lee, Jin-Choon Lee, Byung-Joo Lee

**Affiliations:** 1 Department of Otolaryngology—Head and Neck Surgery, Samsung Changwon Hospital, Sungkyunkwan University School of Medicine, Changwon 51353, Republic of Korea; uuhent@skku.edu (G.C.P.); naronaro@skku.edu (H.P.); 2 Department of Otorhinolaryngology—Head and Neck Surgery, College of Medicine, Pusan National University and Biomedical Research Institute, Pusan National University Hospital, Busan 49241, Republic of Korea; sooyoungbang@pusan.ac.kr (S.-Y.B.); jimin-kim@pusan.ac.kr (J.M.K.); shinsc0810@pusan.ac.kr (S.-C.S.); skydragonone@pusan.ac.kr (Y.-i.C.); 3 Division of Gastroenterology, Department of Medicine, Samsung Changwon Hospital, Sungkyunkwan University School of Medicine, Changwon 51353, Republic of Korea; kwmin.kim@samsung.com; 4 Department of Otorhinolaryngology—Head and Neck Surgery, College of Medicine, Pusan National University and Biomedical Research Institute, Pusan National University Yangsan Hospital, Yangsan 50612, Republic of Korea; sunges@pusan.ac.kr (E.-S.S.); weichwein@naver.com (M.L.); ljc020971@pusan.ac.kr (J.-C.L.)

**Keywords:** obesity, ferroptosis, metabolic-dysfunction-associated steatotic liver disease (MASLD), hepatic steatosis, iron metabolism

## Abstract

Ferroptosis, a form of regulated cell death characterized by lipid peroxidation and iron accumulation, has been implicated in the progression of metabolic-dysfunction-associated steatohepatitis (MASH) in obesity. This study investigated the role of ferroptosis in the development of hepatic steatosis and MASH in obese mice and assessed the therapeutic potential of ferrostatin-1, a ferroptosis inhibitor. C57BL/6J wild-type (n = 8) and ob/ob mice (n = 16) were maintained on a standard chow diet. Mice were divided into three groups that included C57BL/6 (n = 8), ob/ob (n = 8), and ob/ob + ferrostatin-1 (FER) (n = 8), with the latter group receiving an intraperitoneal injection of 5 μM/kg ferrostatin three times per week for eight weeks. Following treatment, serum and tissue samples were collected for analysis. Significant hepatic steatosis and increased lipogenesis markers were observed in ob/ob mice, which were restored to baseline levels in the ob/ob + FER group treated with ferrostatin-1. Elevated oxidative stress was indicated by increased reactive oxygen species (ROS) and malondialdehyde (MDA) levels in the ob/ob group, while glutathione peroxidase 4 (GPX4) activity was significantly reduced. Ferrostatin-1 treatment decreases MDA levels and restores GPX4 activity. Additionally, ferrostatin mitigates iron overload and promotes macrophage polarization from M1 to M2, thereby reducing liver inflammation and fibrosis. Ferrostatin treatment reversed mitochondrial dysfunction in ob/ob mice. Our findings revealed that ferroptosis plays a significant role in the progression of obesity to hepatic steatosis and MASH. Inhibiting ferroptosis using ferrostatin-1 effectively improves liver histology, reduces oxidative stress, normalizes lipogenesis, and modulates macrophage polarization. This study highlights the potential of targeting ferroptosis as a therapeutic strategy for obesity-related liver diseases, warranting further investigation in clinical settings.

## 1. Introduction

Obesity is a chronic disease that can cause various health problems due to excessive fat deposition in the body. In the World Obesity Atlas 2023 report, 38% of the global population is overweight or obese, and this proportion is expected to rise rapidly over time [1]. Obesity is a major contributor to cancer, cardiovascular disease, and early death and significantly affects various organs. One of the significant consequences of obesity is its crucial role in the development of metabolic-dysfunction-associated steatotic liver disease (MASLD) [2,3,4].

MASLD is a broad-spectrum liver disease characterized by fat accumulation in the liver in the absence of alcohol. It starts with hepatic steatosis (HS), a condition in which there are only fat deposits in the liver tissue, and when liver function deteriorates and an inflammatory response occurs, it becomes metabolic-dysfunction-associated steatohepatitis (MASH). When fibrosis worsens and scarring occurs in the tissue, it progresses to hepatofibrosis (HF) and eventually progresses to hepatocellular carcinoma (HCC) [5]. MASLD is also strongly associated with various metabolic diseases such as type 2 diabetes and dyslipidemia. MASLD is known to occur in more than 65% of obese individuals, and its prevalence is rising in parallel with increasing obesity rates [6,7,8,9]. However, the detailed mechanisms underlying the occurrence and progression of MASLD are not yet known. Therefore, no therapeutic drugs have been developed.

Ferroptosis is a ferrous (Fe^2+^)-dependent cell death process that is bioenergetically distinct from apoptosis. Ferroptosis is closely related to several diseases such as oxidative stress, inflammatory responses, and autophagy [10]. Numerous studies have revealed its involvement in the occurrence and progression of various diseases such as stroke, cancer, chronic mental illness, cardiovascular disease, and diabetes [10,11,12]. Excessive intracellular iron accumulation and lipid reactive oxygen species (ROS) are required to initiate ferroptosis. ROS generated by the catalytic action of iron initiate lipid peroxidation and cause ferroptosis [10]. The liver is the primary organ that regulates lipid metabolism and iron homeostasis [13,14]. If adipose tissue accumulates in the liver, lipid ROS levels can increase, and iron accumulation can also occur. Therefore, lipid accumulation and iron imbalance caused by obesity can be considered as conditions that promote ferroptosis in the liver [15,16,17]. Two previous studies demonstrated that ferroptosis plays an important role in the progression and worsening of MASH [18,19]; however, these studies were conducted in mouse models already induced with MASH. Specific research investigating the role of ferroptosis in the development of hepatic steatosis and its progression to MASH has not yet been conducted.

As simple hepatic steatosis progresses to MASH, the incidence of liver cirrhosis increases along with the risk of progression to liver failure and liver cancer. Therefore, prevention of hepatic steatosis and its progression to MASH is critical. Although oxidative stress is thought to play an important role in liver cell damage and the progression to MASH, the exact mechanisms involved in the development of hepatic steatosis and its progression to MASH remain unclear. In the present study, we investigated the role of ferroptosis in the development of hepatic steatosis and its progression to MASH in the context of obesity. Additionally, we aimed to determine whether the modulation of ferroptosis could prevent or facilitate recovery from hepatic steatosis and MASH.

## 2. Materials and Methods

### 2.1. Animals and Experimental Design

The ob/ob mouse model lacks the leptin gene, leading to hyperphagia and obesity, which mimics human metabolic disorders. By using this model, we aim to elucidate the role of ferroptosis in liver disease progression. Six-week-old female C57BL/6J-Jms Slc wild-type mice (n = 8) and six-week-old female C57BL/6J ob/ob mice (n = 16) were used, The purchased from Japan SLC, Inc. (Shizuoka, Japan). After a two-week adaptation period, mice were divided into three groups: C57BL/6 (n = 8), ob/ob (n = 8), and ob/ob + ferrostatin-1 (FER) (n = 8). The mice were maintained on a standard chow diet. The ob/ob + FER group was administered ferrostatin-1 for 8 weeks (intraperitoneal injection, 5 μM/kg/day) three times per week from 8-week ob/ob mice [20]. C57BL/6 and ob/ob mice were injected with equal volumes of normal saline solution using the same injection schedule. The ob/ob group underwent tests at the start of the experiment (ob/ob 8wks) and at 8 weeks (ob/ob 16wks) to observe the progression of fatty liver disease and hepatitis as obesity progressed. Eight weeks after drug injection, the animals were respiratory-anesthetized, blood was collected from the inferior vena cava for biomarker testing, and they were then sacrificed. This study was approved by the Institutional Animal Care and Ethics Committee of our hospital (No. PNUH-2022-200).

### 2.2. Serum and Tissue Collection

Blood samples were extracted from the inferior vena cava using a 1 mL syringe and coagulated for 2 h at room temperature [21]. After centrifugation, serum samples were removed and stored at −80 °C until analysis. Serum aspartate aminotransferase (AST), alanine aminotransferase (ALT), total cholesterol, and blood glucose levels were measured.

### 2.3. Lipid Peroxidation

Lipid peroxidation in the liver tissue was evaluated by measuring malondialdehyde (MDA) levels using a Lipid Peroxidation (MDA) Assay Kit (Abcam, Cambridge, UK) as previously described. Briefly, liver tissue was homogenized in TBA solution, and samples and standards were prepared. These were incubated at 95 °C for 60 min and then cooled in an ice bath for 10 min. The lipid peroxidation assay of malondialdehyde (MDA) with TBA produced TBA-MDA with a colorimetric (532 nm) readout. This assay colorimetrically detects MDA levels as low as 1 nmol/well.

### 2.4. Haematoxylin and Eosin (H&E) Staining

Liver samples were isolated from each rat and fixed overnight in 4% formalin. An automatic tissue processor was used for paraffin embedding (Leica, Wetzlar, Germany; Leica, TP1020, a semi-enclosed benchtop tissue processor) and dispensing (Leica, Wetzlar, Germany; Leica EG1150H, a heated paraffin embedding module). Cross-sections (8-μm thick) of livers were placed on glass slides, and sections were prepared for hematoxylin-eosin (H&E) staining. For staining analyses, the slides were deparaffinized with xylene and then hydrated through a series of washes in 100%, 85%, 75%, and 50% ethanol mixed with water. The central part of the tissue was selected for representative figures using 200× images acquired by a light microscope (Leica, Wetzlar, Germany, Leica DM4000/600M, versatile upright microscope for material analysis).

### 2.5. Real-Time Quantitative Polymerase Chain Reaction (RT-qPCR)

Liver tissue RNA was extracted using TRIzol reagent (Life Technologies, Carlsbad, CA, USA). A reverse transcription kit (Applied Biosystems, Foster City, CA, USA) was used at 42 °C for 60 min and 95 °C for 10 min according to the manufacturer’s protocol. The qPCR was performed according to the SYBR^®^ Green PCR protocol (Applied Biosystems). Gene-specific PCR products were continuously analyzed using an ABI PRISM 7900 HT Sequence Detection System (PE Applied Biosystems, Foster City, CA, USA). Primer sequences were synthesized by Macrogen.

Normalization was performed based on the differences between the target gene cycle thresholds (Ct) and Glyceraldehyde-3-phosphate dehydrogenase (GAPDH) expression level to calculate the Ct:target gene Ct ratio (ΔCt). The primer sequences are listed in Table 1.

### 2.6. Western Blot

The protein samples were separated by sodium dodecyl sulfate-polyacrylamide gel electrophoresis (SDS-PAGE) and transferred onto a nitrocellulose (NC) blotting membrane (Amersham, Taufkirchen, Germany), followed by incubation with 5% phospho-blocker at room temperature for 1 h.

The membrane was incubated overnight at 4 °C with primary antibodies that included anti-LC3B antibody (1:1000), anti-NCOA4 antibody (1:1000), and β-actin antibody. The next day, the NC membrane was incubated with horseradish peroxidase (HRP)-labeled secondary antibodies (1:5000) for 2 h at room temperature and developed using an ECL kit (Amersham, Taufkirchen, Germany).

## 3. Results

We previously conducted research about ferroptosis in the salivary glands. We explored the use of deferoxamine as a ferroptosis inhibitor in addition to ferrostatin-1. Deferoxamine is known to impede intracellular iron accumulation, thus preventing the progression of ferroptosis; however, our previous research did not yield satisfactory results, and the data were inconsistent [22]. Therefore, in this study, we decided to proceed with experiments using only ferrostatin-1.

### 3.1. Body Weight, Food Intake, and Serum Glucose Level

**Figure 1:**
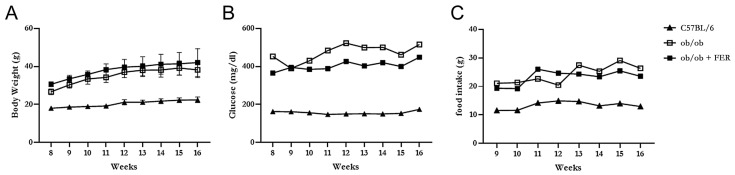
Body weight, food intake, and serum glucose levels in the C57BL/6, ob/ob, and ob/ob + FER groups over 8 weeks. (**A**) Body weight increased to approximately 40 g in the ob/ob and ob/ob + FER groups, while the C57BL/6 group remained at approximately 20 g (*p* < 0.05). (**B**) Serum glucose levels were consistently below 200 mg/dL in C57BL/6, whereas the ob/ob and ob/ob + FER groups exceeded 400 mg/dL (*p* < 0.05). (**C**) Food intake was significantly higher in that ob/ob and ob/ob + FER groups compared to that in the C57BL/6 group (*p* < 0.05).

The C57BL/6, ob/ob, and ob/ob + FER groups were monitored weekly for food intake, body weight, and serum glucose levels for 8 weeks. The C57BL/6 group maintained a relatively stable food intake without significant changes from the start of the experiment through to week 8, with body weight exhibiting a slight increase and remaining in the 20 g range. In contrast, both the ob/ob and ob/ob + FER groups exhibited nearly double the food intake compared to that of the C57BL/6 group, with the body weight gradually increasing to approximately 40 g. Both groups exhibited significant differences in food intake and body weight compared to that of the C57BL/6 group (*p* < 0.05) (Figure 1A,C). The serum glucose level in the C57BL/6 group remained consistently below 200 mg/dL throughout the 8 weeks, whereas the ob/ob and ob/ob + FER groups exhibited significantly higher levels that exceeded 400 mg/dL (*p* < 0.05), with more variability than that of the C57BL/6 group during the same period (Figure 1B). No significant differences in food intake, body weight, or serum glucose levels were observed between the ob/ob and ob/ob + FER groups. These data confirmed that the ob/ob group serves as a suitable obese mouse model, and consistent administration of ferrostatin for 8 weeks did not significantly affect body weight or serum glucose levels, indicating that ferrostatin does not exert a direct influence on obesity.

### 3.2. Lipogenesis and Steatosis in Obese Mice and the Inhibitory Effect of the Ferroptosis Inhibitor

**Figure 2:**
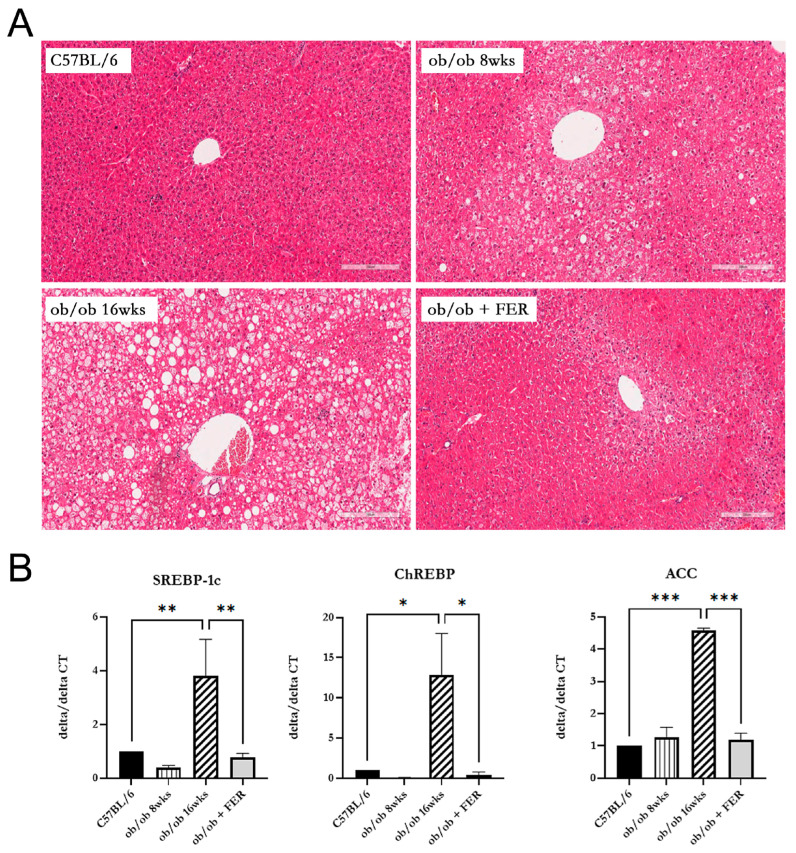
Lipogenesis and steatosis in the livers of the C57BL/6, ob/ob, and ob/ob + FER groups. (**A**) C57BL/6 livers exhibited a normal morphology, while the ob/ob group displayed significant steatosis by week 8. The FER group treated with ferrostatin possessed liver tissue resembling that of C57BL/6, with nearly all lipid droplets eliminated. Scale bar = 200 μm. (**B**) At 8 weeks, SREBP-1c, ChREBP, and ACC levels were lower in the ob/ob group but were significantly increased by 16 weeks. The ob/ob + FER group maintained levels similar to those of the C57BL/6 group, indicating that ferrostatin normalizes lipogenesis and steatosis. * *p* < 0.05, ** *p* < 0.01, *** *p* < 0.001. Columns and error bars represent the mean ± standard deviation.

We compared lipogenesis and steatosis in the livers of the three groups. The liver morphology of C57BL/6 mice was normal. The ob/ob group exhibited pre-existing lipid droplets at the start of the experiment, and significant steatosis was evident by week 8. The ob/ob + FER group, treated with the ferroptosis inhibitor ferrostatin-1 for eight weeks, exhibited liver tissue similar to that of the control C57BL/6 mice, with nearly all lipid droplets eliminated compared to those of the initial ob/ob mice (Figure 2A).

To assess the degree of lipogenesis in the livers of the three groups, we compared the expression levels of the lipogenesis-related markers SREBP-1c, ChREBP, and ACC. At 8 weeks, the expression level of SREBP-1c in the ob/ob group was similar to that of the C57BL/6 group. However, at 16 weeks, the expression of SREBP-1c in the ob/ob group was over three-fold greater than that in the C57BL/6 group (*p* < 0.01). Additionally, the expression of ChREBP in the 16-week ob/ob group was over ten-fold higher compared to the C57BL/6 group (*p* < 0.05). A similar trend was observed for ACC, with the 8-week ob/ob group showing comparable expression levels to the C57BL/6 group, while the 16-week ob/ob group exhibited a more than four-fold increase in expression compared to the C57BL/6 group (*p* < 0.001). Importantly, the ob/ob + FER group maintained levels of SREBP-1c, ChREBP, and ACC similar to those in the normal control C57BL/6 group (Figure 2B).

Comparing the data regarding steatosis and lipogenesis across the three groups, the obese ob/ob model mice initially exhibited no significant differences from the C57BL/6 group. However, by week 16, both steatosis and lipogenesis had progressed significantly, diverging from those in the C57BL/6 group. Remarkably, administration of ferrostatin-1 to ob/ob mice for 8 weeks restored both steatosis and lipogenesis to baseline levels comparable to those in the C57BL/6 group.

### 3.3. Progression of Ferroptosis in Obese Mice and Evaluation of the Efficacy of Ferrostatin for Attenuating Ferroptosis

**Figure 3:**
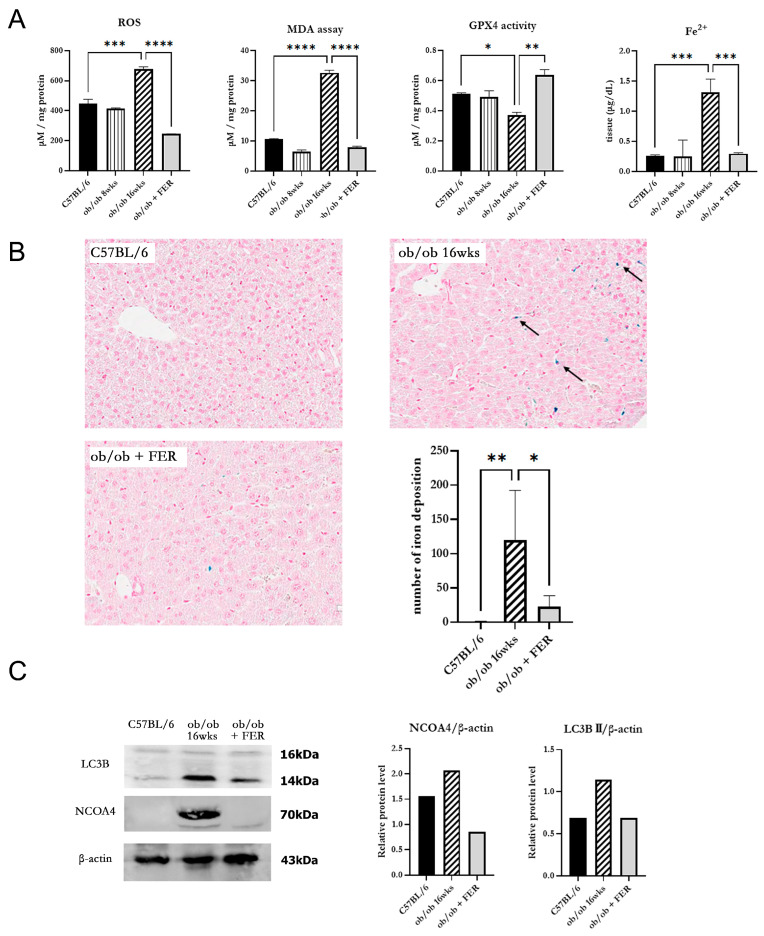
Oxidative stress and iron accumulation in obese mice and the efficacy of ferrostatin treatment. (**A**) Intracellular ROS levels in the ob/ob group were similar to those in the C57BL/6 group at 8 weeks but were significantly increased by 16 weeks. The ob/ob + FER group exhibited lower ROS levels than both the C57BL/6 and ob/ob groups. MDA levels exhibited a three-fold increase at 16 weeks in the ob/ob group compared to levels in the C57BL/6 group, while the ob/ob + FER group returned to normal MDA levels. GPX4 activity decreased significantly in the ob/ob group but was preserved in the ob/ob + FER group. Ferrous iron (Fe^2+^) levels were significantly elevated only in the 16-week ob/ob group. (**B**) Prussian blue staining revealed iron accumulation (arrow) solely in the 16-week ob/ob group (×20). (**C**) According to Western blot analysis, NCOA4 and LC3B expression was present in the 16-week ob/ob group but was reduced in the ob/ob + FER group. * *p* < 0.05, ** *p* < 0.01, *** *p* < 0.001, **** *p* < 0.0001. Columns and error bars represent the mean ± standard deviation.

To investigate changes in oxidative stress due to obesity, we examined intracellular ROS generation in the three groups. The ROS levels in the ob/ob mouse group were similar to those in the C57BL/6 group at 8 weeks but were significantly increased by 16 weeks (*p* < 0.05). Conversely, the ob/ob + FER group demonstrated a significant reduction in ROS levels that were lower than those in the C57BL/6 and 8-week ob/ob groups. MDA, a product of lipid peroxidation, is an indicator of ferroptosis. At 8 weeks, the MDA concentration in ob/ob mice did not differ significantly from that in the C57BL/6 group, but by 16 weeks, it was significantly elevated, exhibiting levels that were three-fold higher than those in the C57BL/6 group (*p* < 0.001). In contrast, ob/ob mice treated with ferrostatin-1 exhibited a return to normal MDA levels (*p* < 0.001). GPX4 is a critical regulator of ferroptosis. The activity of GPX4, a key ferroptosis biomarker, was significantly lower in the 16-week ob/ob group than it was in the C57BL/6 group, while GPX4 activity was significantly higher in the ob/ob + FER group than it was in the ob/ob group (*p* < 0.05) (Figure 3A).

To assess iron overload, another axis of ferroptosis, we measured intracellular ferrous iron (Fe^2+^) levels and used Prussian blue iron staining to detect iron accumulation in the liver tissues of the three groups. The levels of intracellular ferrous iron were similar across the C57BL/6, 8-week ob/ob, and ob/ob + FER groups, with a significant elevation observed only in 16-week ob/ob mice (*p* < 0.001). Prussian blue staining confirmed iron accumulation in tissues exclusively from 16-week ob/ob mice (Figure 3B).

NCOA4 is a selective cargo receptor that mediates the autophagic turnover of ferritin into ferrous iron. NCOA4-mediated ferritinophagy ultimately increases oxidative iron levels and promotes lipid peroxidation. Western blot analysis revealed that LC3B and NCOA4 were expressed in 16-week ob/ob mice, whereas their expression was significantly reduced in the ob/ob + FER group (Figure 3C).

These results indicate that as obesity progresses, intracellular oxidative iron levels increase, driven by NCOA4-mediated ferritinophagy. Additionally, ferrostatin suppressed the expression of NCOA4, thereby attenuating the accumulation of intracellular Fe^2+^.

### 3.4. Effect of Ferrostatin on the Progress of Metabolic-Dysfunction-Associated Steatotic Liver Disease (MASLD) in Obese Mice

**Figure 4:**
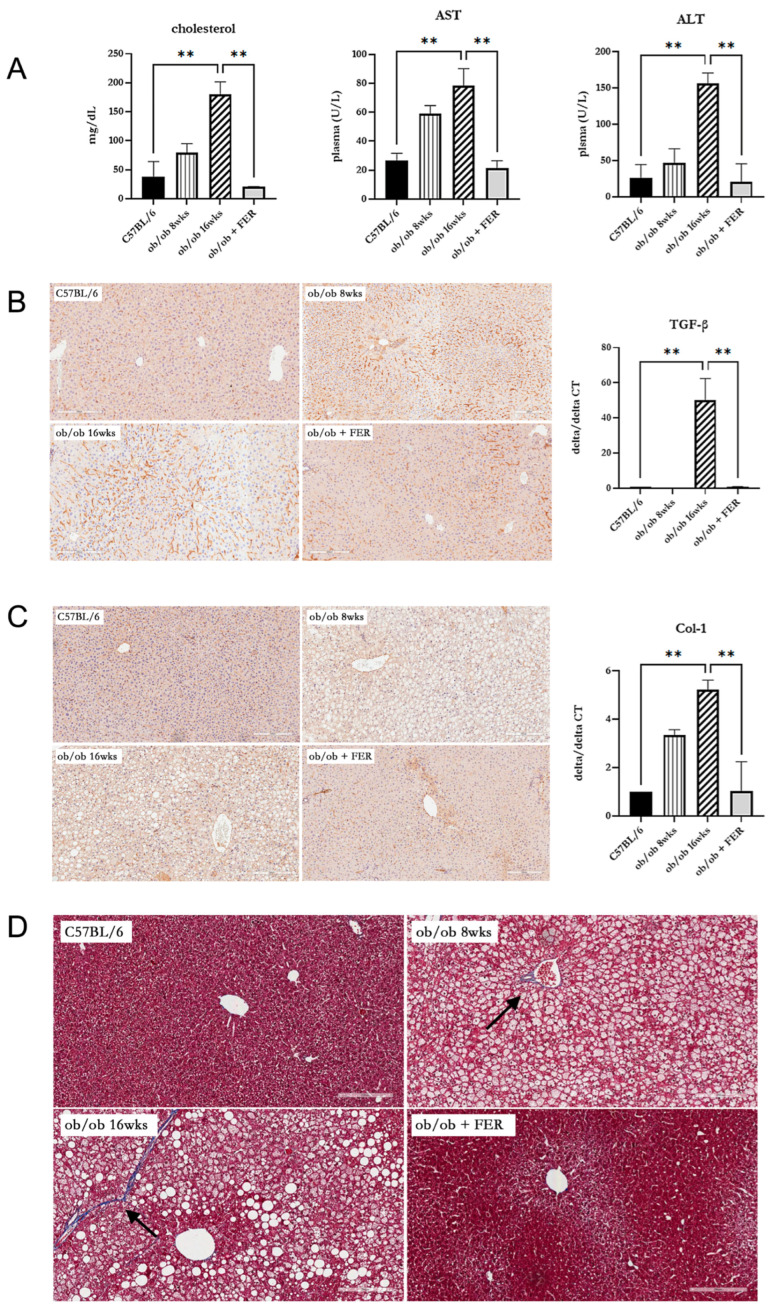
Impact of ferrostatin on liver function, inflammation, and fibrosis in obese mice. (**A**) Serum cholesterol, AST, and ALT levels were significantly higher in the 16-week ob/ob group compared to those in the C57BL/6 group. In contrast, levels in the ob/ob + FER group returned to baseline levels similar to those of the C57BL/6 group. (**B**) TGF-β staining intensity increased in the ob/ob group from 8 to 16 weeks, while it decreased in the ob/ob + FER group. (**C**) Collagen type I expression was higher in the ob/ob group, peaking at 16 weeks, but was restored to baseline levels in the ob/ob + FER group. (**D**) Masson’s trichrome staining revealed no fibrosis in the C57BL/6 group, weak staining in the ob/ob group at 8 weeks, and intensified staining in the ob/ob group at 16 weeks (arrow). No staining was present in the ob/ob + FER group. Scale bar = 200 μm. ** *p* < 0.01. Columns and error bars represent the mean ± standard deviation.

To assess the impact of the duration of obesity on the liver, we evaluated liver function, inflammation, and fibrosis in the liver tissues of the three groups. Serum cholesterol, AST, and ALT measurements revealed that the ob/ob group at 8 weeks exhibited higher levels than C57BL/6 mice, with the 16-week ob/ob group exhibiting even more significant increases. The levels in the ob/ob + FER group were similar to those in the C57BL/6 group (*p* < 0.01) (Figure 4A). These findings suggest that prolonged obesity worsens liver function and that treatment with ferrostatin-1 can normalize liver function, even in the context of long-term obesity. To confirm the inflammatory responses in liver tissues, TGF-β immunohistochemical staining revealed an increased staining intensity from 8 to 16 weeks in the ob/ob group, while staining decreased in the ob/ob + FER group. The mRNA expression of TGF-β was also significantly elevated in the 16-week ob/ob group, but it became restored to baseline levels in the ob/ob + FER group (*p* < 0.01) (Figure 4B). Collagen type I staining also indicated a heightened expression in the ob/ob group compared to that in the C57BL/6 group, with greater levels observed at 16 weeks than at 8 weeks. The ob/ob + FER group returned to levels similar to those observed in the C57BL/6 group. The mRNA expression of collagen type I was also significantly elevated in the 16-week ob/ob group but was restored to baseline levels in the ob/ob + FER group (*p* < 0.01) (Figure 4C). Masson’s trichrome staining, used to assess the degree of fibrosis in liver tissue, exhibited a similar pattern. No staining was observed in the C57BL/6 group, whereas the ob/ob group exhibited weak staining at 8 weeks, which intensified at 16 weeks. No staining was observed in the ob/ob + FER group (Figure 4D).

Collectively, these three immunohistochemical staining results indicate that liver inflammation and fibrosis in obese mice progressed over time, whereas treatment with ferrostatin effectively restored both inflammation and fibrosis to baseline levels.

### 3.5. Induction of the Polarization of M1 to M2 Macrophages by Ferrostatin

**Figure 5:**
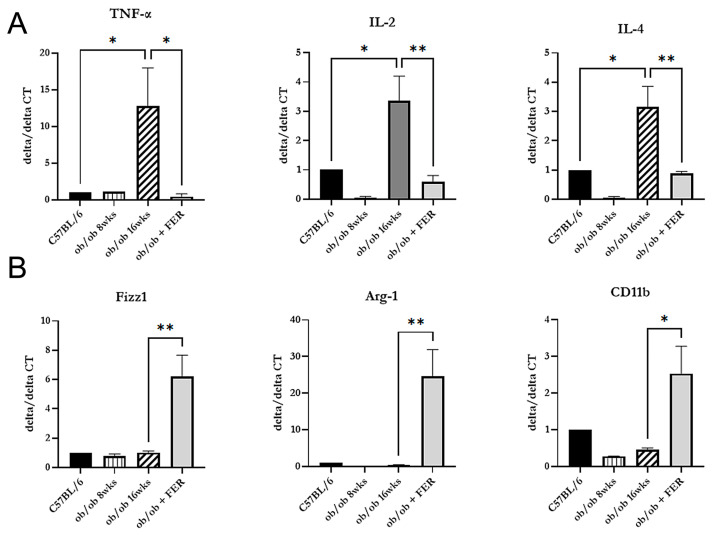
Macrophage polarization changes in response to ferrostatin treatment. Analysis of mRNA expression revealed significantly higher levels of pro-inflammatory cytokines (**A**) in 16-week ob/ob mice compared to those in the other groups, while levels of anti-inflammatory cytokines (**B**) were similar to those in the control group. Ferrostatin treatment in ob/ob mice inhibits M1 cytokines and increases M2 cytokines, promoting a shift from M1 to M2 macrophages. * *p* < 0.05, ** *p* < 0.01. Columns and error bars represent the mean ± standard deviation.

After confirming that inflammation in the liver of obese mice improved with ferrostatin treatment, we investigated the changes in macrophage types across the three groups. We measured the mRNA expression of pro-inflammatory cytokines (TNF-alpha, IL-2, and IL-4) indicative of M1-type macrophages and anti-inflammatory cytokines (Fizz1, Arg-1, and CD11b) produced by M2-type macrophages. The 16-week ob/ob group exhibited significantly higher expression levels of TNF-alpha, IL-2, and IL-4 compared to those of the other groups, while Fizz1, Arg-1, and CD11b exhibited levels similar to those of the C57BL/6 group (*p* < 0.05) (Figure 5A). Conversely, treatment with ferrostatin shifted M1 inflammatory macrophages to M2 anti-inflammatory macrophages in the livers of ob/ob mice by inhibiting TNF-alpha, IL-2, and IL-4 while increasing Fizz1, Arg-1, and CD11b (*p* < 0.05) (Figure 5B).

When correlating these results with the immunohistochemical staining findings presented in Figure 4, we observed that as obesity progressed, M1 macrophages became activated, leading to chronic inflammation and subsequent fibrosis. However, treatment with ferrostatin resulted in the repolarization of M1 to M2 macrophages, facilitating recovery from liver inflammation and fibrosis.

### 3.6. Mitochondrial Dysfunction in Obese Mice

**Figure 6:**
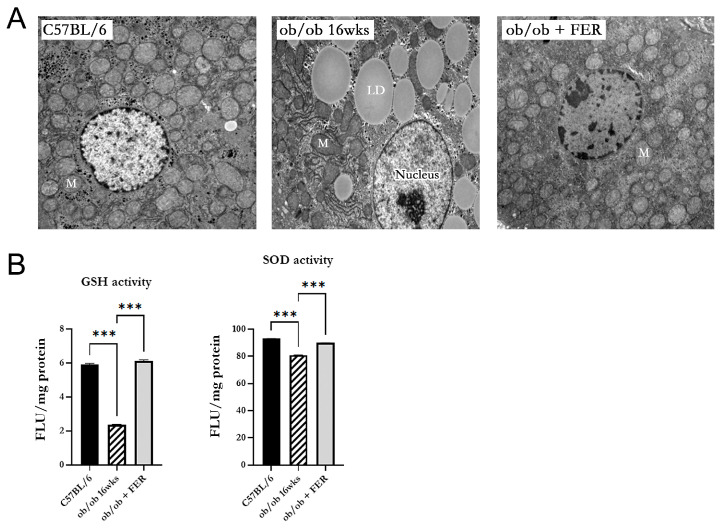
Restoration of mitochondrial structure and function by ferrostatin in obese mice. (**A**) Electron microscopy revealed that mitochondria in the 16-week ob/ob group displayed an increased membrane density, loss of cristae, and contraction compared to the C57BL/6 group. In contrast, the ob/ob + FER group exhibited a restored-to-baseline mitochondrial morphology, with cristae levels comparable to those of the C57BL/6 group. M, mitochondria; LD, lipid droplet (×5000). (**B**) The activities of GSH and SOD significantly decreased in the 16-week ob/ob group but were restored to normal levels in the ob/ob + FER group. *** *p* < 0.001. Columns and error bars represent the mean ± standard deviation.

We investigated the morphology and function of mitochondria in the hepatic cells of obese mice. Electron microscopy of 16-week ob/ob mice revealed mitochondria with an increased membrane density, loss of cristae, and overall contraction compared to those in the C57BL/6 group. In contrast, the ob/ob + FER group exhibited mitochondria with a restored-to-baseline-level membrane structure and cristae comparable to those of the C57BL/6 group (Figure 6A). Additionally, the activities of GSH and SOD that assess mitochondrial function were significantly decreased in 16-week ob/ob mice but were restored to normal levels in the ferrostatin-treated group (*p* < 0.001) (Figure 6B).

## 4. Discussion

In this study, we elucidated the critical role of ferroptosis at each stage of the progression from hepatic steatosis to metabolic-dysfunction-associated steatohepatitis (MASH) in obese mice, revealing that the inhibition of ferroptosis can serve as an effective strategy to prevent the progression of fatty liver disease and MASH. Research on the role of ferroptosis in metabolic liver diseases has recently gained attention [15,16,17,23,24,25]. Due to the characteristics of the liver, where lipid peroxidation occurs readily, the hepatic cellular environment is inherently conducive to ferroptosis. Therefore, ferroptosis significantly contributes to hepatocyte damage and inflammatory responses in MASLD. Studies conducted in MASH mouse models have emphasized that ferroptosis contributes to liver inflammation and fibrosis progression, thereby exacerbating the disease. Additionally, it possesses the potential to delay liver disease progression and improve therapeutic outcomes by regulating ferroptosis [18,19]. However, as previous studies were conducted in mouse models that induced MASH, ferroptosis could only be observed in the process of worsening hepatitis when hepatitis had already occurred, and the role of ferroptosis in the development of hepatic steatosis and hepatitis in obesity remains unknown. There is a definite difference between the starting point of this study and those of previous studies. In this study, we directly observed the process of obesity developing into hepatic steatosis and hepatitis using an ob/ob mouse model that induces obesity due to mutation of the leptin gene. We confirmed for the first time that ferroptosis plays an important role in the occurrence of hepatic steatosis and hepatitis and that this process can be prevented by inhibiting ferroptosis.

In the ob/ob mouse model, ferroptosis is associated with increased oxidative stress and lipid peroxidation, as evidenced by elevated levels of MDA and reduced GPX4 activity. These results align with the existing literature highlighting the role of oxidative stress in liver pathology [18,19]. The significant increase in lipid peroxidation markers in the ob/ob group suggests that as hepatic steatosis progresses, the liver becomes more susceptible to ferroptosis, exacerbating metabolic dysfunction. Importantly, ferrostatin administration mitigated these effects, normalized lipid peroxidation and GPX4 activity, and significantly improved liver histology. These results indicate that the inhibition of ferroptosis can prevent the progression of hepatic steatosis to the more severe stages of MASLD. This finding is crucial, as it suggests a potential therapeutic avenue for managing obesity-related liver diseases.

Furthermore, the results demonstrate a clear relationship between iron overload and ferroptosis. Ferroptosis, a unique form of iron-related cell death, is critically dependent on iron metabolism and is promoted by intracellular iron accumulation [26,27,28]. Consequently, understanding iron metabolism is crucial for understanding the mechanisms underlying ferroptosis. However, previous studies investigating ferroptosis in MASH have not adequately analyzed iron metabolism [18,19]. In the present study, the increased levels of intracellular ferrous iron in ob/ob mice, coupled with the expression of NCOA4, highlighted the role of ferritinophagy in the accumulation of reactive iron species. The reduced expression of NCOA4 following ferrostatin treatment underscores the potential of targeting this pathway to limit oxidative damage and subsequent cell death.

Inflammation observed in the liver tissues of ob/ob mice is indicative of a chronic inflammatory state associated with obesity. During the immune response, M1 macrophages are initially activated to address foreign materials, followed by M2 macrophage activation to facilitate tissue recovery [29]. However, if M1 to M2 polarization does not occur, continuous activation of M1 macrophages leads to inadequate tissue repair and fibrosis. This phenomenon has been observed in various chronic inflammatory diseases [30,31], and previous studies have confirmed an increased M1/M2 ratio in obese mice [32]. Our finding of a polarization shift from M1 to M2 macrophages following ferrostatin treatment suggests that ferroptosis influences the inflammatory microenvironment of the liver. M1 macrophages promote inflammation, whereas M2 macrophages exhibit anti-inflammatory effects. The decrease in pro-inflammatory cytokines along with the increase in anti-inflammatory markers further supports the notion that ferroptosis modulation can have systemic anti-inflammatory effects that are critical for liver health.

In terms of mitochondrial health, ob/ob mice exhibited significant mitochondrial dysfunction characterized by structural abnormalities and decreased enzyme activity. Ferrostatin treatment not only restored mitochondrial morphology but also improved mitochondrial function, suggesting that ferroptosis contributes to mitochondrial impairment in obesity. This restoration of mitochondrial function could play a role in the recovery of liver health and reversal of MASLD [33,34].

The improvement in fibrosis observed in ferrostatin-treated mice, as evidenced by Masson’s trichrome staining, is a significant and encouraging finding in our study. This result suggests that ferroptosis inhibition may not only prevent the progression of fibrosis but can also reverse existing fibrosis in the context of MASLD. The ability to attenuate or reverse fibrosis represents a major breakthrough in the treatment of chronic liver diseases. Although these results are promising, caution should be exercised while interpreting their broader implications. Further research is necessary to fully elucidate the mechanisms by which ferroptosis inhibition affects fibrosis and determine the extent and durability of its antifibrotic effects. Future studies should consider incorporating a quantitative analysis of fibrosis markers such as hydroxyproline content and a gene expression analysis of fibrosis-related genes [35,36]. This study possessed another limitation. It remains unclear whether ferrostatin inhibits all subsequent stages by blocking steatosis and lipogenesis or whether it directly intervenes at each individual stage. More rigorously designed studies are needed to clarify this issue. Additionally, this study can be used to determine whether ferrostatin can prevent progression to subsequent steps by only inhibiting steatosis and lipogenesis or whether it directly intervenes at each individual stage. Finally, in this research, the eight ob/ob mice administered ferrostatin did not show any significant differences in body weight, diet, or other parameters compared to the ob/ob mice that did not receive ferrostatin. Additionally, throughout the observation period, their activity levels remained normal, and no unusual physiological signs were observed. Although potential detrimental effects of ferrostatin-1 have not yet been reported, future studies should also investigate the potential side effects of ferrostatin-1.

**Figure 7:**
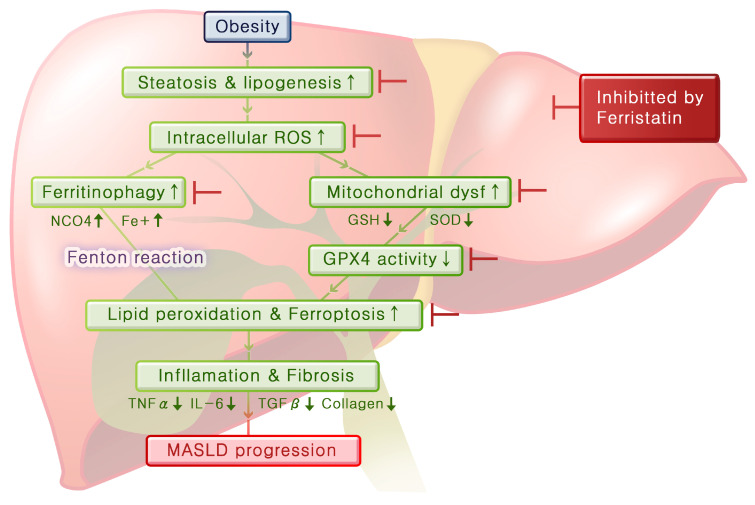
Mechanisms linking obesity to fatty liver disease and hepatitis mediated by ferroptosis. Obesity induces hepatic steatosis and lipogenesis, leading to increased intracellular ROS levels. Elevated oxidative stress levels contribute to mitochondrial dysfunction and ferritinophagy, which decrease GPX4 activity and increase intracellular ferrous iron levels. This signaling cascade promotes lipid peroxidation and initiates ferroptosis. As ferroptosis progresses, liver inflammation worsens, resulting in fibrosis. Importantly, ferrostatin treatment inhibits steatosis and lipogenesis, prevents ferritinophagy and mitochondrial dysfunction, and restores GPX4 activity, ultimately blocking the progression to ferroptosis. Additionally, ferrostatin aids in the recovery of pre-existing inflammation and prevents the further development of fibrosis.

By integrating these experimental results, we can infer the mechanisms that lead from obesity to fatty liver disease and hepatitis (Figure 7). Obesity induces steatosis and lipogenesis in the liver tissue, resulting in increased intracellular ROS levels. Elevated oxidative stress levels lead to mitochondrial dysfunction and ferritinophagy, and this decreases GPX4 activity and increases ferrous iron levels, respectively, thereby promoting lipid peroxidation and initiating ferroptosis. As ferroptosis progresses, inflammation in the liver worsens and fibrosis develops over time. Importantly, ferrostatin administration can inhibit steatosis and lipogenesis and prevent both ferritinophagy and mitochondrial dysfunction. Furthermore, ferrostatin restored GPX4 activity, ultimately blocking the progression of ferroptosis. It can also recover pre-existing inflammation and prevent the progression of fibrosis.

## 5. Conclusions

Our study highlights the dual role of ferroptosis in obesity that contributes to the development and progression of MASLD and represents a viable therapeutic target. Given the increasing prevalence of obesity-related liver diseases, targeting ferroptosis may represent a novel approach for the prevention and treatment of MASLD. Future studies should focus on elucidating the molecular pathways involved in ferroptosis within the liver and exploring the therapeutic potential of ferroptosis inhibitors in clinical settings.

## Figures and Tables

**Table 1:** Sequence of primers.

Gene	Sequence (5′–3′)	
	Forward	Reverse
GAPDH	AGCCCAAGATGCCCTTCAGT	CCGTGTTCCTACCCCCAATG
SREBP-1c	ACGGAGCCATGGATTGCACA	AAGGGTGCAGGTGTCACCTT
ChREBP	CTGGGGACCTAAACAGGAGC	GAAGCCACCCTATAGCTCCC
ACC	ATGGGCGGAATGGTCTCTTTC	TGGGGACCTTGTCTTCATCAT
TGF-β1	GTGTGGAGCAACATGTGGAACTCTA	TTGGTTCAGCCACTGCCGTA
Col1	CCTCAGGGTATTGCTGGACAAC	CAGAAGGACCTTGTTTGCCAGG
TNF-α	GGTGCCTATGTCTCAGCCTCTT	GCCATAGAACTGATGAGAGGGAG
IL-4	GGTCACAGGAGAAGGGACGCC	TGCGAAGCACCTTGGAAGCCC
Fizz1	CTG CCC TGC TGG GAT GAC T	CAT CAT ATC AAA GCT GGG TTC TCC
Arg-1	GGA ATC TGC ATG GGC AAC CTG TGT	AGG GTC TAC GTC TCG CAA GCC A
CD11b	CCA CAG TTC ACA CTT CTT TCA G	TGT CCA GAT TGA AGC CAT GA

## Data Availability

Data used to support the findings of this study are available from the corresponding author upon request.
